# Juzentaihoto Failed to Augment Antigen-Specific Immunity but Prevented Deterioration of Patients' Conditions in Advanced Pancreatic Cancer under Personalized Peptide Vaccine

**DOI:** 10.1155/2013/981717

**Published:** 2013-06-10

**Authors:** Shigeru Yutani, Nobukazu Komatsu, Satoko Matsueda, Munehiro Yoshitomi, Takahisa Shirahama, Akira Yamada, Kyogo Itoh, Tetsuro Sasada

**Affiliations:** ^1^Department of Immunology and Immunotherapy, Kurume University School of Medicine, Kurume 830-0011, Japan; ^2^Research Center for Innovative Cancer Therapy, Kurume University, Kurume 830-0011, Japan; ^3^Department of Surgery, Kurume University School of Medicine, Kurume 830-0011, Japan

## Abstract

Juzentaihoto (JTT) is a well-known Japanese herbal medicine, which has been reported to modulate immune responses and enhance antitumor immunity in animal models. However, it is not clear whether JTT has similar effects on humans. In particular, there is little information on the effects of JTT in antigen-specific immunity in cancer patients. Here we conducted a randomized clinical study to investigate whether combined usage of JTT could affect antigen-specific immunity and clinical findings in advanced pancreatic cancer patients undergoing personalized peptide vaccination (PPV), in which HLA-matched vaccine antigens were selected based on the preexisting host immunity. Fifty-seven patients were randomly assigned to receive PPV with (*n* = 28) or without (*n* = 29) JTT. Unexpectedly, JTT did not significantly affect cellular or humoral immune responses specific to the vaccine antigens, which were determined by antigen-specific interferon-*γ* secretion in T cells and antigen-specific IgG titers in plasma, respectively. Nevertheless, JTT prevented deterioration of patients' conditions, such as anemia, lymphopenia, hypoalbuminemia, plasma IL-6 elevation, and reduction of performance status, which are frequently observed in advanced cancers. To our knowledge, this is the first clinical study that examined the immunological and clinical effects of JTT in cancer patients undergoing immunotherapy in humans.

## 1. Introduction

Juzentaihoto (JTT) is a well-known Kampo (Japanese herbal) medicine, which consists of 10 different herbs and has been used as a supplementary therapy in patients with various types of chronic diseases/symptoms, such as fatigue, loss of appetite, night sweats, circulatory problems, and anemia [1]. JTT has also been frequently used for cancer patients, since it was reported to have anti-tumor effects [1–7] and diminish the side effects caused by cancer treatments, such as chemotherapy and radiotherapy [8–12]. In addition, JTT was shown to possess immune-modulating properties, such as enhancement of phagocytosis, cytokine production, antibody production, and NK, NKT, and T-cell functions, in animal experiments [1–7, 13–21]. However, only limited information is available on the immunological and clinical effects of JTT in humans. 

Pancreatic cancer, the fourth largest cause of cancer death in the world, is one of the most aggressive cancers [22, 23]. Although there have been substantial advances in the therapeutic modalities for pancreatic cancer, including systemic chemotherapies using gemcitabine (GEM), S-1 (tegaful, gimeracil, and oteracil potassium), and/or molecular-targeted agents, the prognosis of advanced pancreatic cancer patients still remains dismal [22, 23]. Therefore, development of new therapeutic approaches, including immunotherapy, is needed.

We have developed a novel immunotherapeutic approach, personalized peptide vaccination (PPV), in which HLA-matched peptides were selected and administered, based on the pre-existing host immunity before vaccination [24–28]. Recent clinical trials of PPV have demonstrated feasibility and safety of this new therapeutic approach in various types of advanced cancers [24–28]. For example, in our previous clinical trials, immune responses boosted by vaccination were well associated with overall survival (OS) in advanced pancreatic cancer patients undergoing PPV in combination with GEM as the first-line therapy [28]. In the current study, we conducted a randomized phase II study of PPV to investigate whether combined usage of JTT could show immunological and/or clinical effects in advanced pancreatic cancer patients undergoing PPV.

## 2. Patients and Methods

### 2.1. Patients

Patients with pathological and/or clinical diagnosis of pancreatic cancer, who were refractory to conventional treatments, such as surgery, chemotherapy, and radiotherapy, were eligible for inclusion in the current study, if they showed positive IgG responses to at least 2 of the 31 different vaccine candidate peptides, as reported previously [24–28]. Other inclusion criteria were as follows: age of more than 20 years; an Eastern Cooperative Oncology Group (ECOG) performance status of 0 or 1; positive status for the HLA-A2, -A24, -A3 supertype (A3, A11, A31, or A33), or -A26; expected life expectancy of at least 12 weeks; and adequate hematologic, hepatic, and renal function. Exclusion criteria included pulmonary, cardiac, or other systemic diseases; an acute infection; a history of severe allergic reactions; regular use of herbal medicines; pregnancy or nursing; and other inappropriate conditions for enrollment as judged by clinicians. The protocol was approved by the Kurume University Ethical Committee and was registered in the UMIN Clinical Trials Registry (UMIN 000006295). After a full explanation of the protocol, a written informed consent was obtained from all patients before enrollment.

### 2.2. Clinical Protocol

This was an open-label, randomized phase II study. The patients were randomly assigned to receive PPV with or without oral administration of JTT (PPV plus JTT group versus PPV alone group), according to age and performance status. The primary and secondary objectives were to compare cellular and humoral immune responses to the vaccine antigens and safety between the PPV plus JTT group and the PPV alone group, respectively. Thirty-one peptides, whose safety and immunological effects had been confirmed in previously conducted clinical studies [24–28], were employed for vaccination (12 peptides for HLA-A2, 14 peptides for HLA-A24, 9 peptides for HLA-A3 supertype (A3, A11, A31, or A33), and 4 peptides for HLA-A26) (Supplementary Table 1) (see Supplementary Material available online at http://dx.doi.org/10.1155/2013/981717). The peptides were prepared under the conditions of Good Manufacturing Practice (GMP) by PolyPeptide Laboratories (San Diego, CA, USA) and the American Peptide Company (Vista, CA, USA).

The peptides for vaccination to individual patients were selected in consideration of the pre-existing host immunity before vaccination, by assessing the titers of IgG specific to each of the 31 different vaccine candidates, as reported previously [24–28]. A maximum of 4 peptides (3 mg/each peptide), which were selected based on the results of HLA typing and peptide-specific IgG titers, in mixture with incomplete Freund's adjuvant (Montanide ISA51; Seppic, Paris, France), were subcutaneously administered once a week for 6 consecutive weeks. In the PPV plus JTT group, JTT (TJ-48, 15 mg/day; Tsumura Co., Tokyo, Japan) was orally administered for 35 days during the first cycle of 6 vaccinations. After the first cycle of 6 vaccinations, up to 4 vaccine peptides were reselected according to the titers of peptide-specific IgG and administered every 2 weeks. The vaccine peptides were re-selected at every cycle of 6 vaccinations until the discontinuation of PPV. Adverse events were monitored according to the National Cancer Institute Common Terminology Criteria for Adverse Events (CTCAE) version 4.0. Complete blood counts and serum biochemistry tests were performed before and after every cycle of 6 vaccinations.

### 2.3. Measurement of T-Cell Responses to the Vaccine Peptides

T-cell responses specific to the vaccine peptides were evaluated by interferon (IFN)-*γ* ELISPOT assay (MBL, Nagoya, Japan). Briefly, peripheral blood mononuclear cells (PBMCs) (2 × 10^5^ cells/well) were cultured in U-bottomed 96-well microculture plates (Nunc, Roskilde, Denmark) with 200 *μ*L of medium (OpTmizer T-Cell Expansion SFM; Invitrogen, Carlsbad, CA, USA) containing 10% FBS (MP Biologicals, Solon, OH, USA), IL-2 (20 IU/mL; AbD Serotec, Kidlington, UK), and each peptide (10 *μ*M). Half of the medium was replaced with new medium containing the corresponding peptides (20 *μ*M) at day 3. After incubation for the following 4 days, the cells were harvested and tested for their ability to produce IFN-*γ* in response to the corresponding specific peptides. The cells were also tested for IFN-*γ* production in response to negative control peptides from human immunodeficiency virus (HIV), which might activate nonspecific immune cells, including non-specific CD8 or CD4 T cells and NK cells. IFN-*γ* secretion after 18-hour incubation was determined by ELISPOT assay with an ELISPOT reader (ImmunoSpot S5 Versa Analyzer; Cellular Technology Ltd., Shaker Heights, OH, USA). All assays were carried out in quadruplicate. The two-tailed Student's *t*-test was used for statistical evaluation. Antigen-specific T-cell responses were considered positive, when the spot numbers in response to the specific peptides were significantly higher (*P* < 0.05) than those in response to the control HIV peptides, which were supposed to reflect the numbers of immune cells nonspecifically producing IFN-*γ*. Peptide-specific T-cell responses were shown as the differences between the spot numbers per 1 × 10^5^ PBMCs in response to the specific peptides and those in response to the control peptides.

### 2.4. Measurement of Humoral Immune Responses to the Vaccine Peptides

The humoral immune responses specific to the vaccine peptides were determined by peptide-specific IgG titers using a bead-based multiplex assay with the Luminex 200 system (Luminex, Austin, TX, USA), as reported previously [29]. In brief, plasma (×100 diluted) was incubated with 100 *μ*L of peptide-coupled color-coded beads for 1.5 hours at 30°C, followed by washing and incubation with 100 *μ*L of biotinylated goat anti-human IgG (Vector Laboratories, Burlingame, CA, USA) for 1 hour at 30°C. The beads were washed and incubated with 100 *μ*L of streptavidin-PE (Invitrogen) for 30 min at 30°C. After washing, the fluorescence of the beads was detected using the Luminex 200 system. If peptide-specific IgG titers in the postvaccination plasma were more than 2-fold higher than those in the prevaccination plasma, the changes were considered to be significant. If a significant increase was observed in at least one of the vaccine peptides, the antigen-specific humoral immune response was considered to be augmented.

### 2.5. Measurement of Laboratory Markers

ELISA kits were used to measure serum amyloid A (SAA) (Invitrogen), IL-6 (eBioscience, San Diego, CA, USA), IL-18 (MBL), and C-reactive protein (CRP), IL-12 and TGF-*β*1 (R&D systems, Minneapolis, MN, USA). Bead-based multiplex assays were used to measure Th1/Th2 cytokines, including IFN-*γ*, IL-2, IL-4, IL-5, and IL-10 (Human Th1/Th2 5-Plex, Invitrogen), with the Luminex 200 system (Luminex). Frozen plasma samples were thawed, diluted, and assayed in duplicate in accordance with the manufacturer's instructions. The mean of duplicate samples was used for statistical analysis.

Free-radical elective evaluator (Wismerll, Tokyo, Japan) was used to measure biological antioxidant potential (BAP) and derivatives of reactive oxidative metabolites (d-ROM), an index of oxidative stress. Frozen plasma samples were thawed, diluted, and assayed in accordance with the manufacturer's instruction.

### 2.6. Flow Cytometric Analysis of a Suppressive Immune Cell Subset in PBMCs

A suppressive immune cell subset, myeloid-derived suppressor cells (MDSCs), in PBMCs was examined by flow cytometry. For analysis of MDSCs, PBMCs (0.5 × 10^6^) were incubated for 30 min at 4°C with monoclonal antibodies (mAbs) against lineage markers (CD3, CD14, CD19, and CD56), CD33, and HLA-DR. After washing, the samples were run on a FACSCanto II (BD biosciences, San Diego, CA, USA), and data were analyzed using the Diva software (BD biosciences). All mAbs were purchased from Biolegend (San Diego, CA). Granulocytic MDSCs were identified as CD33 positive in the cell subset negative for both the lineage markers and HLA-DR. Monocytic MDSCs were identified as CD14 positive and HLA-DR negative. The frequency of MDSCs in the mononuclear cell gate defined by the forward scatter and side scatter was calculated.

### 2.7. Statistical Methods

The Wilcoxon signed-rank test, Student's *t*-test, the chi-square test, or Fisher's exact test was used to compare differences between measurements. OS was calculated from the first date of peptide vaccination until the date of death or the last date when the patient was known to be alive. Curves for OS were estimated by the Kaplan-Meier method, and the log-rank test was conducted for the comparison of survival curves. Two-sided *P* values of <0.05 were considered as statistically significant. All statistical analyses were conducted using the JMP version 10.0 software (SAS Institute Inc., Cary, NC, USA).

## 3. Results

### 3.1. Patients' Characteristics

Between September 2011 and December 2012, a total of 57 advanced pancreatic cancer patients, who were refractory to conventional treatments, were enrolled in this study. The patients were randomly assigned in a 1 : 1 ratio to receive PPV with or without oral administration of JTT (PPV plus JTT, *n* = 28; PPV alone, *n* = 29). The demographic and baseline disease characteristics of the enrolled patients are given in Table 1. There were no significant differences between the two groups in the clinicopathological characteristics, including age, gender, performance status, HLA-type, clinical stage, location of the main tumor, and numbers of previous chemotherapy regimen(s). The median number of vaccinations was 9 (range 3–17) in the PPV plus JTT group and 10 (range 3–18) in the PPV alone group. Five and 2 patients did not complete the first cycle of 6 vaccinations due to disease progression in the PPV plus JTT group and the PPV alone group, respectively. In the PPV plus JTT group, PPV was combined with GEM (*n* = 10), S-1 (*n* = 5), GEM and S-1 (*n* = 7), or other combinations of chemotherapeutic agents (*n* = 2). Four patients received PPV alone because they could not tolerate chemotherapy. In the PPV alone group, PPV was combined with GEM (*n* = 13), S-1 (*n* = 7), GEM and S-1 (*n* = 8), or other combination of chemotherapeutic agents (*n* = 1). 

### 3.2. Adverse Events

Adverse events occurring in the patients are listed in Table 2. The most frequent adverse event was injection site reactions in both groups. Severe adverse events (grade 3 or grade 4) were as follows: gamma-glutamyl transpeptidase (GGT) increase (*n* = 2), alkaline phosphatase (ALP) increase (*n* = 1), glucose intolerance (*n* = 1), ascites (*n* = 1), and biliary tract infection (*n* = 1) in the PPV plus JTT group; GGT increase (*n* = 3), thrombocytopenia (*n* = 1), anorexia (*n* = 1), and pain (*n* = 1) in the PPV alone group. There were no significant differences in the overall rates of adverse events between the PPV plus JTT group and the PPV alone group. According to assessment by the independent safety evaluation committee in this trial, all of these severe adverse events were due to cancer progression or other causes, such as side effects related to combined chemotherapies, rather than to the administration of peptide vaccines or JTT.

### 3.3. Cellular and Humoral Immune Responses to the Vaccine Peptides

Cellular and humoral immune responses specific to the vaccine peptides were analyzed in blood samples before and after the first cycle of vaccination (Supplementary Table 2 and Supplementary Table 3). Since 5 and 2 patients did not complete the first cycle of 6 vaccinations due to disease progression in the PPV plus JTT group and the PPV alone group, respectively, post-vaccination samples of these patients were unavailable. 

T-cell responses to the vaccine peptides were measured by IFN-*γ* ELISPOT assay with PBMCs. PBMCs were available for this assay in 27 and 22 patients before and after the first cycle of vaccination in the PPV plus JTT group, respectively (Supplementary Table 2). In this group, antigen-specific T-cell responses were detectable in 2 of 27 patients (7.4%) and 5 of 22 patients (22.7%) before and after vaccination, respectively. In the PPV alone group, PBMCs were available in 28 and 26 patients before and after the first cycle of vaccination, respectively (Supplementary Table 3). In this group, antigen-specific T-cell responses were detectable in 4 of 28 patients (14.3%) and 11 of 26 patients (42.3%) before and after vaccination, respectively. There were no significant differences between the PPV plus JTT group and the PPV alone group in the antigen-specific T-cell responses both before and after vaccination (*P* = 0.669 and *P* = 0.260, resp.) (Table 3). 

In addition, the humoral immune responses specific to the vaccine peptides were determined by peptide-specific IgG titers using a bead-based multiplex assay. Plasma samples both before and after the first cycle of vaccination were available in 23 and 27 patients in the PPV plus JTT group and the PPV alone group, respectively (Supplementary Table 2 and Supplementary Table 3). The IgG responses specific to at least one of the vaccine peptides were augmented in 10 of 23 patients (43.5%) and in 10 of 27 patients (37.0%) in the PPV plus JTT group and the PPV alone group, respectively. There was no significant difference in the augmentation of antigen-specific humoral immune responses between the two groups (*P* = 0.643) (Table 3).

### 3.4. Clinical Outcome

**Figure 1:**
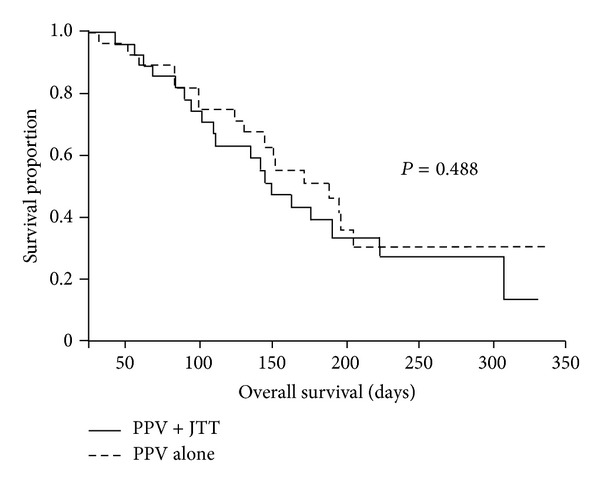
Kaplan-Meier survival analysis in advanced pancreatic cancer patients undergoing PPV with or without JTT. Curves for overall survival were estimated in the PPV plus JTT group (*n* = 28) and the PPV alone group (*n* = 29) by the Kaplan-Meier method, and a difference between survival curves was statistically analyzed using the log-rank test.

All the 57 patients were analyzed for OS. Median followup was 148 (95% confidence interval (CI), 123 to 176) days. The median survival times (MST) from the first vaccination were 148 (95% CI, 109 to 222) days and 187 (95% CI, 129 to undefined) days in the PPV plus JTT group and the PPV alone group, respectively. There was no significant difference in OS between groups (*P* = 0.488, log-rank test) (Figure 1). 

In the PPV alone group, 6 of 29 patients showed reduced ECOG performance status during or after the first cycle of vaccination. In contrast, in the PPV plus JTT group, performance status was reduced during or after the first cycle of vaccination in only 3 of 28 patients. A significant change in performance status was observed between before and after (or during) vaccination in the PPV alone group (*P* = 0.0156, paired Wilcoxon signed-rank test) but not in the PPV plus JTT group (*P* = 0.125, paired Wilcoxon signed-rank test).

### 3.5. Laboratory Markers

**Figure 2:**
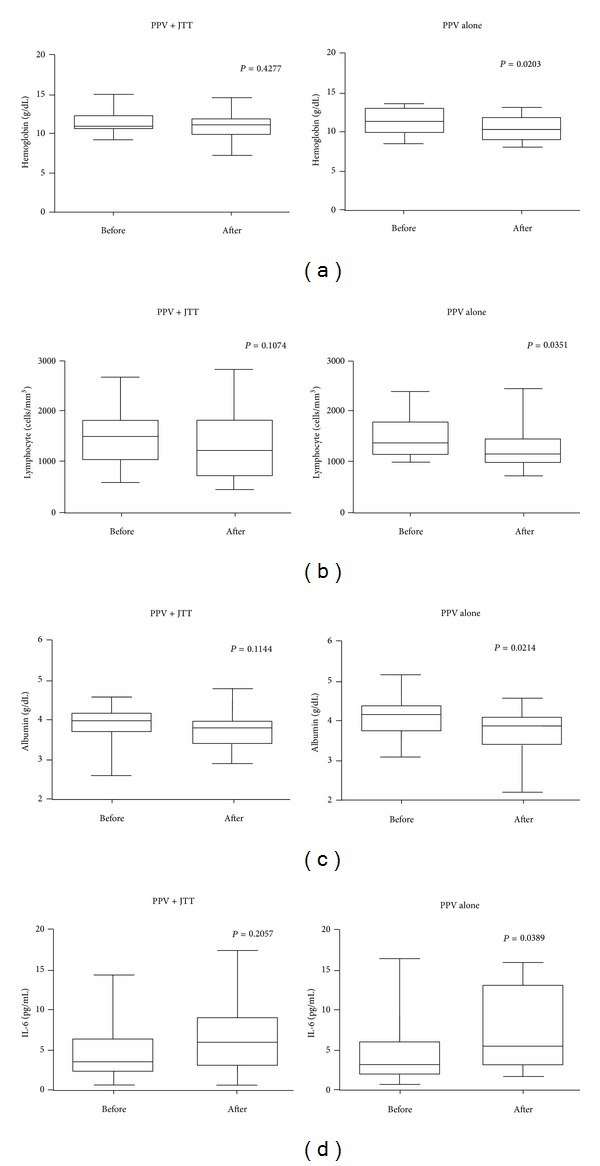
Laboratory markers before and after vaccination in advanced pancreatic cancer patients undergoing PPV with or without JTT. Laboratory markers were compared between before and after the first cycle of 6 vaccinations in the PPV plus JTT group (*n* = 23) and the PPV alone group (*n* = 27) by the paired Wilcoxon signed-rank test. The levels of hemoglobin (a), lymphocyte counts (b), albumin (c), and IL-6 (d) in peripheral blood before and after vaccination are shown. The results are represented by box-and-whiskers graphs. The box plots show median and interquartile range. The whiskers go down to the lowest value and up to the highest value.

Laboratory data both before and after the first cycle of vaccination were available in 23 and 27 patients in the PPV plus JTT group and the PPV alone group, respectively. Complete blood counts and serum biochemistry tests were compared between the two groups. There were no significant differences in complete blood counts, such as hemoglobin and lymphocyte counts, and serum biochemistry tests, such as albumin, total bilirubin, and creatinine, before vaccination (Table 4). In the PPV alone group, hemoglobin, lymphocyte counts, and albumin were significantly decreased after the first cycle of vaccination, whereas they did not change significantly after vaccination in the PPV plus JTT group (Figures 2(a), 2(b), and 2(c)). Of note, these results were consistent, even if 4 patients without combined chemotherapies were excluded from the PPV plus JTT group for statistical analysis. This finding suggested that combined usage of JTT prevented the decrease in hemoglobin, lymphocyte counts, and albumin in pancreatic cancer patients undergoing PPV. 

In addition, other markers, including cytokines (IL-2, IL-4, IL-5, IL-6, IL-10, IL-12, IL-18, IFN-*γ*, and TGF-*β*1), inflammation markers (CRP and SSA), and oxidative stress markers (d-ROM and BAP), were compared between the PPV plus JTT group and the PPV alone group. There were no significant differences between the two groups in all of these markers examined before vaccination (Table 4). Inflammatory cytokine IL-6 was significantly increased after the first cycle of vaccination in the PPV alone group, but not in the PPV plus JTT group, suggesting that combined usage of JTT inhibited plasma IL-6 elevation in pancreatic cancer patients undergoing PPV (Figure 2(d)). There were no significant changes in other markers between before and after vaccination in the PPV plus JTT group or in the PPV alone group (data not shown). In addition, there were no significant changes in suppressive immune cell subsets, granulocytic and monocytic MDSCs, in PBMCs between before and after vaccination in the PPV plus JTT group or in the PPV alone group (data not shown).

## 4. Discussion

JTT is a well-known Kampo (Japanese herbal) medicine and has been shown to possess immune-modulating and antitumor properties in animal experiments [1–7, 13–21]. However, only limited information is available on the immunological and clinical effects of JTT in cancer patients. To our knowledge, this is the first clinical study that examined the immunological and clinical effects of JTT in cancer patients undergoing immunotherapy in humans.

JTT has been reported to modulate antigen-specific adoptive immune responses in mice [2, 15]. For example, Dai et al. demonstrated that oral administration of JTT induced cytotoxic T cells specific to tumor cells and prevent tumor development in the RET-transgenic mouse model [2]. Iijima et al. reported that JTT induced Th1-skewed immune responses and Th1-dependent antibody responses in aged mice [15]. However, the current study showed that combined usage of JTT did not significantly affect cellular or humoral immune responses to the vaccine antigens after PPV. JTT has also been shown to enhance production of cytokines, such as IL-12 and IL-18, in mice [17, 18]. But, in the current study, there were no significant differences in production of several different cytokines, except IL-6, between the PPV plus JTT group and the PPV alone group. Furthermore, there were no significant differences in suppressive immune cell subsets, granulocytic and monocytic MDSCs [30, 31], in PBMCs between the two groups. Based on our results, combined usage of JTT had no significant immune-modulating effects in advanced cancer patients undergoing PPV, in disagreement with the results of previous animal experiments. In addition, although JTT was reported to inhibit immune cell-mediated oxidative stress [6, 19], the current study showed no significant effects of JTT in redox status, which was determined by oxidative stress markers (d-ROM and BAP) in plasma, in advanced cancer patients undergoing PPV.

Several previous reports demonstrated that JTT showed antitumor effects through various mechanisms [1–7]. Ohnishi et al. showed that oral administration of JTT before tumor inoculation resulted in dose-dependent inhibition of liver metastasis of colon 26-L5 carcinoma cells [5]. Matsuda et al. also reported that oral administration of JTT before tumor cell injection significantly inhibited lung metastasis of B16 melanoma cells in mice [4]. In addition, in humans, JTT supplementation was shown to result in considerable improvement in intrahepatic recurrence-free survival in hepatocellular carcinoma (HCC) patients after surgical treatment [6]. Although these results suggested the preventive effects of JTT in tumor development in mice and humans, the therapeutic effects of this agent for advanced stage of tumors are not well defined. The current study showed that combined usage of JTT conferred no survival benefits in patients with pancreatic cancer undergoing PPV. 

Combined usage of PPV and JTT was well tolerated. The most frequent adverse event was injection site reactions, and all of the severe adverse events observed were due to cancer progression or other causes rather than to the vaccinations or JTT administration. Of note, JTT administration induced some beneficial effects in pancreatic cancer patients undergoing PPV. Although the patients treated with PPV alone showed decrease in hemoglobin, lymphocyte counts, and albumin after vaccination possibly due to side effects of combined chemotherapies and/or malnutrition mediated by disease progression, those treated with PPV in combination with JTT maintained a stable level of these factors, as previously suggested [1, 12, 32]. Consistent with these findings, a significant change in performance status was observed between before and after (or during) vaccination in the PPV alone group but not in the PPV plus JTT group. These results suggest that JTT has the potential to prevent deterioration of patients' conditions without severe adverse events even in advanced cancer patients undergoing immunotherapy. Other clinical data, such as patients' quality of life (QOL), were unavailable in this study, but they might be worthy of assessment in future clinical trials. 

It should also be noted that the elevation of the pro-inflammatory cytokine IL-6 was inhibited by combined usage of JTT. IL-6 is a multifunctional cytokine that regulates various aspects of immune responses, acute phase reactions, and hematopoiesis. In particular, IL-6 has been reported to be deeply involved in inflammation associated with cancer development and progression [33, 34]. Indeed, there have been many studies describing the correlation between IL-6 elevation and poor prognosis in various types of cancers, including pancreas cancer [35–38]. In addition, IL-6 has recently been reported to be one of the critical cytokines for inducing suppressive immune cell subsets, such as MDSCs and Th17, which are known to negatively affect anti-tumor immunity [39–41]. Therefore, the inhibitory effect of JTT on IL-6 elevation might be beneficial for controlling cancer progression.

## 5. Conclusion

In summary, we for the first time examined the immunological and clinical effects of JTT in cancer patients undergoing cancer vaccination in humans. Our randomized clinical trial of PPV with or without JTT suggested that combined usage of JTT revealed a potential to prevent deterioration of patients' conditions but had no effects in antigen-specific immunity in advanced pancreatic cancer patients. Since all of the enrolled patients had rapidly progressive advanced tumors, it might be possible that JTT supplementation for a limited, short period was not sufficient to elicit beneficial immune responses in the treated patients. A next step of randomized clinical trials of PPV with or without JTT would thus be recommended in cancer patients in the adjuvant setting or in those with more slowly growing tumors.

## Supplementary Material

Supplementary Table 1. The list of peptide candidates employed for personalized peptide vaccination.
Supplementary Table 2. Humoral and cellular immune responses to the vaccine antigens before and after vaccination in each patient of the PPV plus JTT group. The humoral immune responses specific to the vaccine peptides were determined by peptide-specific IgG titers in plasma before and after the first cycle of vaccination using a bead-based multiplex assay. T cell responses specific to the vaccine peptides were evaluated by IFN-**γ** ELISPOT assay with PBMCs before and after the first cycle of vaccination.
Supplementary Table 3. Humoral and cellular immune responses to the vaccine antigens before and after vaccination in each patient of the PPV alone group. The humoral immune responses specific to the vaccine peptides were determined by peptide-specific IgG titers in plasma before and after the first cycle of vaccination using a bead-based multiplex assay. T cell responses specific to the vaccine peptides were evaluated by IFN-**γ** ELISPOT assay with PBMCs before and after the first cycle of vaccination.

## Figures and Tables

**Table 1:** Characteristics of the enrolled patients.

Factor	PPV + JTT (*n* = 28)	PPV alone (*n* = 29)	*P* value
Age (years)			0.389
Median (range)	66 (50–83)	65 (45–79)	
Gender			0.922
Male	18	19	
Female	10	10	
Performance status			0.706
0	19	22	
1	9	7	
HLA type			0.753
A24	18	15	
A2	12	13	
A3 supertype	10	17	
A26	5	7	
Clinical stage			0.845
IV	19	20	
Recurrence	9	9	
Location of the main tumor			0.182
Head	6	12	
Body-tail	22	17	
Number of previous chemotherapy regimens			0.843
0	1	1	
1	11	13	
2	13	10	
>3	3	5	
Number of vaccinations			0.443
Median (range)	9 (3–17)	10 (3–18)	
Combination chemotherapy			0.640
None	4	0	
Gemcitabine	10	13	
S-1	5	7	
Gemcitabine + S-1	7	8	
Others	2	1	

**Table 2:** Adverse events.

Adverse events	PPV + JTT (*n* = 28)				Total (%)	PPV alone (*n* = 29)				Total (%)
	G1	G2	G3	G4		G1	G2	G3	G4	
Injection site reaction	15				15 (54%)	20				20 (69%)
Blood/bone marrow										
Leukopenia	3	2			5 (18%)	4				4 (14%)
Lymphopenia	3	2			5 (18%)	3	1			4 (14%)
Anemia	3	4			7 (25%)	2	4			6 (21%)
Thrombocytopenia	1	1			2 (7%)	2		1		3 (10%)
Laboratory										
AST increased	2	1			3 (11%)	4	2			6 (21%)
ALT increased	4	1			5 (18%)	3	2			5 (17%)
Bilirubin increased		1			1 (4%)	1				1 (3%)
GGT increased	1	8	1	1	11 (39%)	1	4	3		8 (28%)
ALP increased	2	1	1		4 (14%)	1	2			3 (10%)
Creatinine increased	1				1 (4%)					0 (0%)
Hypoalbuminemia	6	1			7 (25%)	6	2			8 (28%)
Glucose intolerance			1		1 (4%)					0 (0%)
Hyponatremia		1			1 (4%)	2				2 (7%)
Hyperkalemia		1			1 (4%)	1				1 (3%)
Gastrointestinal disorders										
Nausea	1				1 (4%)	1	1			2 (7%)
Diarrhea	2				2 (7%)					0 (0%)
Constipation		1			1 (4%)		1			1 (3%)
Abdominal pain	1				1 (4%)	1	2			3 (10%)
Gastroesophageal reflux disease		1			1 (4%)					0 (0%)
Ascites			1		1 (4%)					0 (0%)
Biliary tract infection			1		1 (4%)		1			1 (3%)
Anorexia		3			3 (11%)	1	1	1		3 (10%)
Fever		1			1 (4%)	3				3 (10%)
Pain		2			2 (7%)	2		1		3 (10%)
Edema limbs	1				1 (4%)		2			2 (7%)
Insomnia					0 (0%)		1			1 (3%)
Rash acneiform					0 (0%)	1				1 (3%)

**Table 3:** Cellular and humoral immune responses to the vaccine antigens.

	PPV + JTT	PPV alone	*P* value
Cellular immune responses to the vaccine antigens*			
Before vaccination	2/27 (7.4%)	4/28 (14.3%)	0.669
After vaccination	5/22 (22.7%)	11/26 (42.3%)	0.260
Humoral immune responses to the vaccine antigens^†^			
Augmented	10/23 (43.5%)	10/27 (37.0%)	0.643

*Antigen-specific T-cell responses were evaluated by IFN-*γ* ELISPOT assay before and after the first cycle of vaccination.

^†^Antigen-specific IgG titers in plasma were evaluated before and after the first cycle of vaccination. If peptide-specific IgG titers in the postvaccination plasma were more than 2-fold higher than those in the prevaccination plasma in at least one of the vaccine peptides, the antigen-specific humoral immune response was considered to be augmented.

**Table 4:** Laboratory markers in peripheral blood before vaccination.

Factor	PPV + JTT (*n* = 28)	PPV alone (*n* = 29)	*P* value
Hemoglobin (g/dL)	11.2 ± 1.4*	11.4 ± 1.6	0.4821
Lymphocyte count (/mm^3^)	1469.8 ± 482.6	1493.3 ± 409.8	0.8732
Albumin (g/dL)	3.9 ± 0.4	4.1 ± 0.5	0.0895
Creatinine (mg/dL)	1.05 ± 1.90	0.72 ± 0.20	0.6791
Total bilirubin (mg/dL)	0.646 ± 0.473	0.583 ± 0.309	0.7829
IL-2 (pg/mL)	6.17 ± 4.45	4.92 ± 4.42	0.3800
IL-4 (pg/mL)	5.247 ± 15.169	0.662 ± 2.117	0.3160
IL-5 (pg/mL)	0.938 ± 3.887	0.098 ± 0.314	0.8965
IL-6 (pg/mL)	5.037 ± 3.786	4.612 ± 4.089	0.5134
IL-10 (pg/mL)	0.000 ± 0.000	0.062 ± 0.284	0.3415
IL-12 (pg/mL)	0.711 ± 0.793	0.637 ± 0.686	0.5433
IL-18 (pg/mL)	580.9 ± 269.5	571.5 ± 236.6	0.9731
IFN-*γ* (pg/mL)	2.87 ± 5.48	2.29 ± 6.66	0.4495
TGF-*β*1 (ng/mL)	5.68 ± 3.08	5.01 ± 1.87	0.7278
C-reactive protein (mg/dL)	1.90 ± 3.50	1.30 ± 1.92	0.2015
Serum amyloid A (*μ*g/mL)	100.66 ± 75.47	69.31 ± 81.49	0.1505
d-ROM (U.CARR)^†^	267.6 ± 51.4	242.2 ± 86.5	0.2424
BAP^‡^ (*μ*mol/L)	973.3 ± 261.0	979.3 ± 183.1	0.7442

*Values are means ± standard deviations.

^†^d-ROM: derivatives of reactive oxidative metabolites: U.CARR, Carratelli unit (1 Carratelli unit = 0.8 mg H_2_O_2_/L).

^‡^BAP: biological antioxidant potential.
