# Supplementary material for: Juzentaihoto Failed to Augment Antigen-Specific Immunity but Prevented Deterioration of Patients' Conditions in Advanced Pancreatic Cancer under Personalized Peptide Vaccine
Source: Evid Based Complement Alternat Med. 2013 Jun 10;2013:981717. doi: 10.1155/2013/981717 (PMC3691906; doi:10.1155/2013/981717)
Supplement: Supplementary file 1 — Supplementary Table 1. The list of peptide candidates employed for personalized peptide vaccination. Supplementary Table 2. Humoral and cellular immune responses to the vaccine antigens before and after vaccination in each patient of the PPV plus JTT group. The humoral immune responses specific to the vaccine peptides were determined by peptide-specific IgG titers in plasma before and after the first cycle of vaccination using a bead-based multiplex assay. T cell responses specific to the vaccine peptides were evaluated by IFN-γ ELISPOT assay with PBMCs before and after the first cycle of vaccination. Supplementary Table 3. Humoral and cellular immune responses to the vaccine antigens before and after vaccination in each patient of the PPV alone group. The humoral immune responses specific to the vaccine peptides were determined by peptide-specific IgG titers in plasma before and after the first cycle of vaccination using a bead-based multiplex assay. T cell responses specific to the vaccine peptides were evaluated by IFN-γ ELISPOT assay with PBMCs before and after the first cycle of vaccination. [file 981717.f1.pdf]

SUPPLEMENTARY TABLE 1: Peptide candidates for cancer vaccination.

Symbol for peptide	Protein	Position of peptide	Amino acid sequence	HLA type
CypB-129	Cyclophilin B	129-138	KLKHYGPGWV	A2, A3sup[a]
Lck-246	p56Lck	246-254	KLVERLGAA	A2
Lck-422	p56Lck	422-430	DVWSFGILL	A2, A3sup
MAP-432	ppMAPkkk	432-440	DLLSHAFFA	A2, A26
WHSC2-103	WHSC2	103-111	ASLDSDPWV	A2, A3sup, A26
HNRPL-501	HNRPL	501-510	NVLHFFNAPL	A2, A26
UBE-43	UBE2V	43-51	RLQEWCSVI	A2
UBE-85	UBE2V	85-93	LIADFLSGL	A2
WHSC2-141	WHSC2	141-149	ILGELREKV	A2
HNRPL-140	HNRPL	140-148	ALVEFEDVL	A2
SART3-302	SART3	302-310	LLQAEAPRL	A2
SART3-309	SART3	309-317	RLAEYQAYI	A2
SART2-93	SART2	93-101	DYSARWNEI	A24
SART3-109	SART3	109-118	VYDYNCHVDL	A24, A3sup, A26
Lck-208	p56Lck	208-216	HYTNASDGL	A24
PAP-213	PAP	213-221	LYCESVHNF	A24
PSA-248	PSA	248-257	HYRKWIKDTI	A24
EGFR-800	EGF-R	800-809	DYVREHKDNI	A24
MRP3-503	MRP3	503-511	LYAWEPSFL	A24
MRP3-1293	MRP3	1293-1302	NYSVRYRPGL	A24
SART2-161	SART2	161-169	AYDFLYNYL	A24
Lck-486	p56Lck	486-494	TFDYLRSVL	A24
Lck-488	p56Lck	488-497	DYLRSVLEDF	A24
PSMA-624	PSMA	624-632	TYSVSFDSL	A24
EZH2-735	EZH2	735-743	KYVGIEREM	A24
PTHrP-102	PTHrP	102-111	RYLTQETNKV	A24
SART3-511	SART3	511-519	WLEYYNLER	A3sup
SART3-734	SART3	734-742	QIRPIFSNR	A3sup
Lck-90	p56Lck	90-99	ILEQSGEWWK	A3sup
Lck-449	p56Lck	449-458	VIQNLERGYR	A3sup
PAP-248	PAP	248-257	GIHKQKEKSR	A3sup

[*]A3sup; HLA-A3 supertype (A3, A11, A31, and A33)

SUPPLEMENTARY TABLE 2: Humoral and cellular immune responses to the vaccine antigens in the PPV plus JTT group.

Patient No.	Peptide	IgG response*		CTL response†	
		Before	After	Before	After
1	SART2-93	128	NA‡	0	NA
	SART3-109	34	NA	0	NA
	Lck-486	136	NA	0	NA
	Lck-488	42	NA	0	NA
2	Lck-246	682	484	0	0
	ppMAPkkk-432	47	252	0	2245
	WHSC2-141	21	11	0	0
	SART3-302	234	23768	0	1260
3	SART3-734	123	83	0	0
	Lck-90	26	17	0	0
	PAP-248	163	145	0	0
	WHSC2-103	25	16	0	0
4	Lck-246	136	114	0	0
	HNRPL-501	378	289	0	0
	UBE2V-85	18996	19986	0	0
	WHSC2-141	274	255	0	0
5	SART2-93	48	73	0	0
	EGF-R-800	30	25	0	0
	Lck-486	26	427	0	695
	Lck-488	198	321	0	0
6	SART2-93	122	167	0	1172
	SART3-109	85	104	0	0
	PSA-248	278	14773	0	0
	MRP3-503	96	6830	0	1902
7	Lck-246	191	195	0	0
	SART3-302	1556	3767	0	0
	Lck-488	56	62	0	0
	PTH-rP-102	194	670	0	0
8	Lck-246	266	265	0	0
	HNRPL-501	131	52	0	0
	WHSC2-141	2020	965	0	0
	SART3-309	288	277	0	0
9	Lck-488	845	904	0	0
	PTHrP-102	630	493	0	0
	SART3-734	221	241	0	0
	WHSC2-103	156	180	0	0
10	Lck-208	527	NA	0	NA
	PAP-213	1795	NA	0	NA
	Lck-488	758	NA	0	NA
	PTHrP-102	137	NA	0	NA
11	SART3-734	23146	4846	0	0
	Lck-90	27	54	0	0
	PAP-248	23	13	0	0
	WHSC2-103	30	40	0	0
12	SART2-93	23	NA	0	NA

	Lck-488	15	NA	0	NA
	PAMA-624	42	NA	0	NA
	WHSC2-103	27	NA	0	NA
13	Lck-246	40	37	0	0
	WHSC2-103	20	18	0	0
	HNRPL-501	40	32	109	0
	SART3-302	3374	24642	0	0
14	SART3-734	106	72	0	0
	Lck-90	12	0	0	0
	Lck-449	109	0	0	0
	WHSC2-103	117	72	0	0
15	SART2-93	55	59	0	0
	EGF-R-800	35	33	0	0
	Lck-486	32	45	0	0
	Lck-488	56	59	0	0
16	Lck-246	211	209	0	1245
	HNRPL-501	134	91	0	0
	SART3-302	141	138	0	0
	Lck-488	47	53	0	0
17	Lck-90	219	178	0	0
	PSA-248	3528	18216	0	0
	EGF-R-800	128	177	0	0
	PSMA-624	50	42	0	0
18	CypB-129	61	49	0	0
	SART2-93	108	128	0	0
	Lck-486	70	60	0	0
	Lck-488	123	119	0	0
19	Lck-246	87	NA	0	NA
	SART3-302	4309	NA	0	NA
	SART2-93	107	NA	0	NA
	PSA-248	86	NA	0	NA
20	SART3-109	49	40	0	0
	SART3-734	2237	1985	0	0
	WHSC2-103	79	49	0	0
	HNRPL-501	37	38	0	0
21	WHSC2-103	744	622	0	0
	HNRPL-501	888	762	0	0
	WHSC2-141	1935	1555	0	0
	PAP-248	267	340	0	0
22	CypB-129	99	277	0	0
	HNRPL-501	3162	5051	0	0
	SART3-511	212	355	0	0
	SART3-734	219	857	0	0
23	SART2-93	67	NA	0	NA
	SART2-161	47	NA	31	NA
	Lck-488	52	NA	0	NA
	ppMAPkkk-432	88	NA	0	NA
24	SART2-93	46	73	0	0
	Lck-488	19	25	0	0
	PTHrP-102	1106	1027	0	0
	PSA-248	41	200	0	0

25	SART2-93	101	119	0	0
	EGF-R-800	588	534	0	0
	Lck-488	81	82	0	0
	Lck-90	59	59	0	0
26	SART2-93	48	62	0	0
	MRP3-1293	3517	3979	0	0
	Lck-486	59	53	0	0
	Lck-90	87	84	0	0
27	SART2-93	261	269	0	1565
	Lck-488	348	418	0	0
	SART3-511	5162	6111	0	0
	SART3-734	460	448	0	0
28	SART2-93	96	88	NA	NA
	PSA-248	38	163	NA	NA
	Lck-486	24	38	NA	NA
	Lck-488	64	45	NA	NA

*Values indicate the fluorescence intensity unit (FIU) of plasma IgG reactive to the corresponding peptides before and after the first cycle of vaccination. If peptide-specific IgG titers in the post-vaccination plasma were more than 2-fold higher than those in the pre-vaccination plasma, the antigen-specific humoral immune response was considered to be augmented. The augmented IgG responses are underlined.

†Values indicate the number of spots per 10^5 PBMCs reactive to the corresponding peptides by IFN-γ ELISPOT assay before and after the first cycle of vaccination. The augmented T cell responses are underlined.

‡NA, not assessed

SUPPLEMENTARY TABLE 3: Cellular and humoral immune responses to the vaccine antigens in the PPV alone group.

Patient No.	Peptide	IgG response*		CTL response†	
		Before	After	Before	After
1	MRP3-1293	203	77	0	429
	PTH-rP-102	309	229	0	0
	SART3-734	504	294	0	0
	PAP-248	682	289	298	0
2	SART2-93	42	44	0	0
	PSA-248	158	555	0	178
	Lck-488	52	68	0	188
	SART3-734	158	229	0	399
3	Lck-246	141	118	0	0
	WHSC2-103	1497	1398	0	0
	UBE2V-43	92	107	0	0
	PAP-248	49	58	0	0
4	SART2-93	91	87	0	0
	SART3-109	77	89	0	0
	Lck-486	39	33	0	0
	Lck-488	27	25	0	0
5	SART3-734	36	23	0	0
	Lck-449	20	10	0	0
	PAP-248	23	11	0	0
	WHSC2-103	86	50	0	0
6	SART2-93	2969	2739	0	190
	EGF-R-800	1611	1466	111	0
	MRP3-1293	1285	1386	0	205
	Lck-488	3082	2943	0	627
7	SART3-734	546	3913	0	0
	Lck-449	141	138	0	0
	PAP-248	113	1121	0	0
	HNRPL-501	110	631	0	0
8	SART3-302	133	164	0	0
	SART2-93	82	98	0	0
	SART3-109	2360	3602	0	130
	Lck-488	71	94	0	0
9	SART2-93	306	NA‡	0	NA
	Lck-488	116	NA	0	NA
	PSMA-624	78	NA	0	NA
	HNRPL-501	85	NA	26	NA
10	SART2-93	319	315	0	0
	Lck-486	69	297	0	0
	Lck-488	91	94	0	0
	PTH-rP-102	764	9512	0	0
11	MRP3-1293	61	59	0	0
	Lck-486	54	416	0	205
	Lck-488	104	126	0	0
	SART3-734	164	206	0	0
12	WHSC2-103	43	59	0	0

	SART3-302	25	44	0	0
	SART3-734	1066	1385	0	0
	Lck-90	51	67	0	0
13	Lck-246	42	51	0	1432
	UBE2V-85	490	17412	0	100
	WHSC2-141	52	238	0	1511
	SART3-734	120	128	0	0
14	SART3-734	1159	5223	88	0
	Lck-449	341	0	119	0
	PAP-248	103	125	0	0
	WHSC2-103	193	0	0	0
15	SART2-93	78	86	0	0
	Lck-488	60	69	0	0
	SART3-511	84	2879	0	0
	SART3-734	1394	818	0	0
16	HNRPL-501	96	88	0	0
	SART3-302	300	347	0	360
	EGF-R-800	831	802	0	0
	Lck-488	137	160	0	0
17	MRP3-1293	617	591	0	0
	Lck-488	541	483	0	0
	Lck-449	570	594	0	0
	WHSC2-103	365	335	0	0
18	HNRPL-501	46	36	0	0
	SART2-93	44	49	0	407
	Lck-208	165	148	0	0
	Lck-488	44	37	0	0
19	CypB-129	79	83	0	0
	WHSC2-103	57	84	0	0
	HNRPL-501	32	40	0	0
	SART3-309	36	50	0	0
20	Lck-246	192	243	0	84
	HNRPL-501	305	102	0	126
	UBE2V-43	247	108	0	168
	SART3-309	204	201	0	0
21	WHSC2-103	48	36	0	0
	WHSC2-141	168	238	0	0
	HNRPL-140	89	68	0	0
	SART3-309	88	73	0	193
22	SART3-109	5564	5397	0	0
	SART3-511	44	46	0	0
	Lck-90	17	22	0	0
	WHSC2-103	24	19	0	0
23	SART3-734	131	808	0	0
	Lck-90	41	38	0	0
	PAP-248	296	3056	0	0
	ppMAPkkk-432	792	506	0	0
24	SART2-93	586	NA	0	NA
	Lck-486	114	NA	0	NA
	Lck-488	129	NA	0	NA
	Lck-90	137	NA	0	NA

25	Lck-246	83	94	0	0
	WHSC2-103	76	77	0	0
	WHSC2-141	235	238	0	0
	SART2-93	93	119	0	0
26	Lck-246	194	348	0	156
	ppMAPkkk-432	511	574	0	701
	WHSC2-141	82	178	0	145
	SART3-302	3864	10792	0	0
27	SART3-309	139	144	0	0
	SART3-109	315	408	0	0
	SART3-511	197	142	0	0
	SART3-734	1194	1252	0	0
28	Lck-488	391	639	0	0
	PTHrP-102	1507	1738	0	0
	SART3-734	1184	2127	0	0
	WHSC2-103	13750	15291	0	0
29	SART3-511	104	91	NA	NA
	SART3-734	120	13167	NA	NA
	Lck-90	56	136	NA	NA
	CypB-129	59	57	NA	NA

*Values indicate the fluorescence intensity unit (FIU) of plasma IgG reactive to the corresponding peptides before and after the first cycle of vaccination. If peptide-specific IgG titers in the post-vaccination plasma were more than 2-fold higher than those in the pre-vaccination plasma, the antigen-specific humoral immune response was considered to be augmented. The augmented IgG responses are underlined.

†Values indicate the number of spots per 10^5 PBMCs reactive to the corresponding peptides by IFN-γ ELISPOT assay before and after the first cycle of vaccination. The augmented T cell responses are underlined.

‡NA, not assessed
